# Composition, richness and nestedness of gallery forest bird assemblages in an Amazonian savanna landscape: lessons for conservation

**DOI:** 10.7717/peerj.12529

**Published:** 2021-12-01

**Authors:** Joandro Pandilha, José Júlio de Toledo, Luis Cláudio Fernandes Barbosa, William Douglas Carvalho, Jackson Cleiton de Sousa, José Maria Cardoso da Silva

**Affiliations:** 1 Programa de Pós-Graduação em Biodiversidade Tropical, Universidade Federal do Amapá, Macapá, Amapá, Brazil; 2 Departamento de Meio Ambiente e Desenvolvimento, Universidade Federal do Amapá, Macapá, Amapá, Brazil; 3 Conservation International-Brazil, Belém, Pará, Brazil; 4 Programa de Pós-Graduação em Biodiversidade e Meio Ambiente, Universidade Federal da Grande Dourados, Dourados, Mato Grosso do Sul, Brazil; 5 Department of Geography and Sustainable Development, University of Miami, Coral Gables, Florida, United States

**Keywords:** Amazon, Riparian forests, Ecology, Communities, Biogeography, Landscape ecology, Sustainability, Brazil, Neotropical, South America

## Abstract

Gallery forests are important to the maintenance of a substantial portion of the biodiversity in neotropical savanna regions, but management guidelines specific to this forest type are limited. Here, we use birds as study group to assess if: (1) functional traits can predict the abundance and occupancy of forest species within a savanna landscape, (2) habitat structures influence the taxonomic, functional, and phylogenetic diversity of forest assemblages, and (3) less diverse gallery forest assemblages are a nested subset of more diverse assemblages living near continuous forests. Then, we propose strategies on how gallery forests can be managed to maintain their species assemblages amidst the fast expansion of human activities across tropical savanna landscapes. We studied 26 sites of gallery forests in an Amazonian savanna landscape and found that: (1) habitat specificity is the only functional trait that predicts species abundance and occupancy across a landscape; (2) phylogenetic diversity is negatively correlated with understory foliage density; (3) the percentage of forests and savannas around sites is positively correlated with both phylogenetic and functional diversity; (4) increasing human activities around gallery forest negatively influences taxonomic and functional diversity; and (5) forest bird assemblages are not distributed at random across the landscape but show a nested pattern caused by selective colonization mediated by habitat filtering. Our combined findings have three implications for the design of conservation strategies for gallery forest bird assemblages. First, maintaining the connectivity between gallery forests and adjacent continuous forests is essential because gallery forest bird assemblages are derived from continuous forest species assemblages. Second, because most species use the savanna matrix to move across the landscape, effectively managing the savanna matrices where gallery forests are embedded is as important to maintaining viable populations of forest bird species as managing the gallery forest themselves. Third, in savanna landscapes planned to be used for agriculture production, protecting gallery forests alone is not enough. Instead, gallery forests should be protected with surrounding savanna buffers to avoid the detrimental effects (edge effects and isolation) of human activities on their biodiversity.

## Introduction

Neotropical savannas regions are globally threatened due to commercial agricultural expansion and silviculture (Silva & Bates, 2002; Carvalho et al., 2019). These regions are home to thousands of endemic species, many of which are at risk due to the lack of adequate protected area systems (Klink & Machado, 2005; Mustin et al., 2017; Carvalho & Mustin, 2017). Neotropical savanna regions are composed of landscapes where the matrix is dominated by open and semi-open upland savannas, intersected by corridors of tall (up to 25 m), evergreen gallery forests that occur naturally as relatively narrow strips (usually no more than 500 m in width) along watercourses (Silva, 1996; Kellman, Tackaberry & Rigg, 1998; Veneklaas et al., 2005; Pennington & Ratter, 2006). These gallery forests, in turn, maintain most of the species of savanna regions even though they occupy only a small portion of them (Redford & Fonseca, 1986; Silva, 1995; Oliveira-Filho & Ratter, 1995; Silva & Santos, 2005).

Gallery forests are not isolated, as they form large dendritic networks following the rivers (Eiten, 1972; Oliveira-Filho & Ratter, 1995; Silva, 1996). Usually, savanna rivers flow towards adjacent areas covered by continuous forests. Thus, gallery forests have been considered conduits that facilitate the colonization of savanna regions by species that have the center of their ranges in the adjacent forest areas (Redford & Fonseca, 1986; Oliveira-Filho & Ratter, 1995; Silva, 1996). However, biogeographic studies have shown that most forest species were not able to colonize gallery forests, and the ones that did so do not extend their ranges deep into savannas (Oliveira-Filho & Ratter, 1995; Silva, 1996). This general pattern suggests that some attributes of gallery forests can also constrain the expansion of forest species across savanna regions.

In general, the impact of a barrier on a species depends on the barrier permeability, which can be defined as the degree by which a barrier constrains a species’ range expansion. The barrier permeability, in turn, is a function of the interactions between barrier attributes and the characteristics (or functional traits) of the organism. Forman (1995) suggests that because forest corridors, such as gallery forests, are narrow, structurally distinct, and have large edge effects (a barrier attribute), they are not able to maintain viable populations of several forest interior species. Thus, their species assemblages are expected to be dominated by generalist species with good dispersal ability (two functional traits). This hypothesis has received support from studies on riparian forest corridors that were created due to human interventions on the landscapes (*e.g*., Metzger, Bernacci & Goldenberg, 1997; Lees & Peres, 2008; Seaman & Schulze, 2010; de Oliveira Ramos & dos Anjos, 2014; Lindsey, Bochio & Anjos, 2019), but it has never been formally tested in natural forest corridors, such as gallery forests. If Forman’s hypothesis is correct and the distributions of gallery forest species across savanna landscapes are indeed determined by a combination of habitat attributes and inter-specific differences in habitat specificity and dispersal ability, then local assemblages formed by such species are expected to show a nested distribution pattern, in which less diverse assemblages are a nested subset of more diverse assemblages living close to continuous forests (Ulrich, Almeida-Neto & Gotelli, 2009; Ulrich & Almeida-Neto, 2012).

In this paper, we combine ecological field data, satellite image analysis, phylogenetic information, and novel statistical techniques to respond to three main questions: (1) Can functional traits predict the abundance and occupancy of forest bird species within savanna landscapes? (2) Do habitat attributes influence the taxonomic, functional, and phylogenetic diversity of forest bird assemblages across a savanna landscape? (3) Are less diverse gallery forest bird assemblages nested subsets of more diverse assemblages? We used birds as the study group because they are diverse, well-known taxonomically, and sensitive to environmental gradients (Sodhi et al., 2011; Sekercioglu, Wenny & Whelan, 2016). Our ultimate aim was to identify general ecological patterns and use these patterns to propose strategies on how gallery forests can be managed to maintain their species assemblages amidst the fast expansion of human activities across tropical savanna landscapes.

## Materials and Methods

### Study landscape

**Figure 1:**
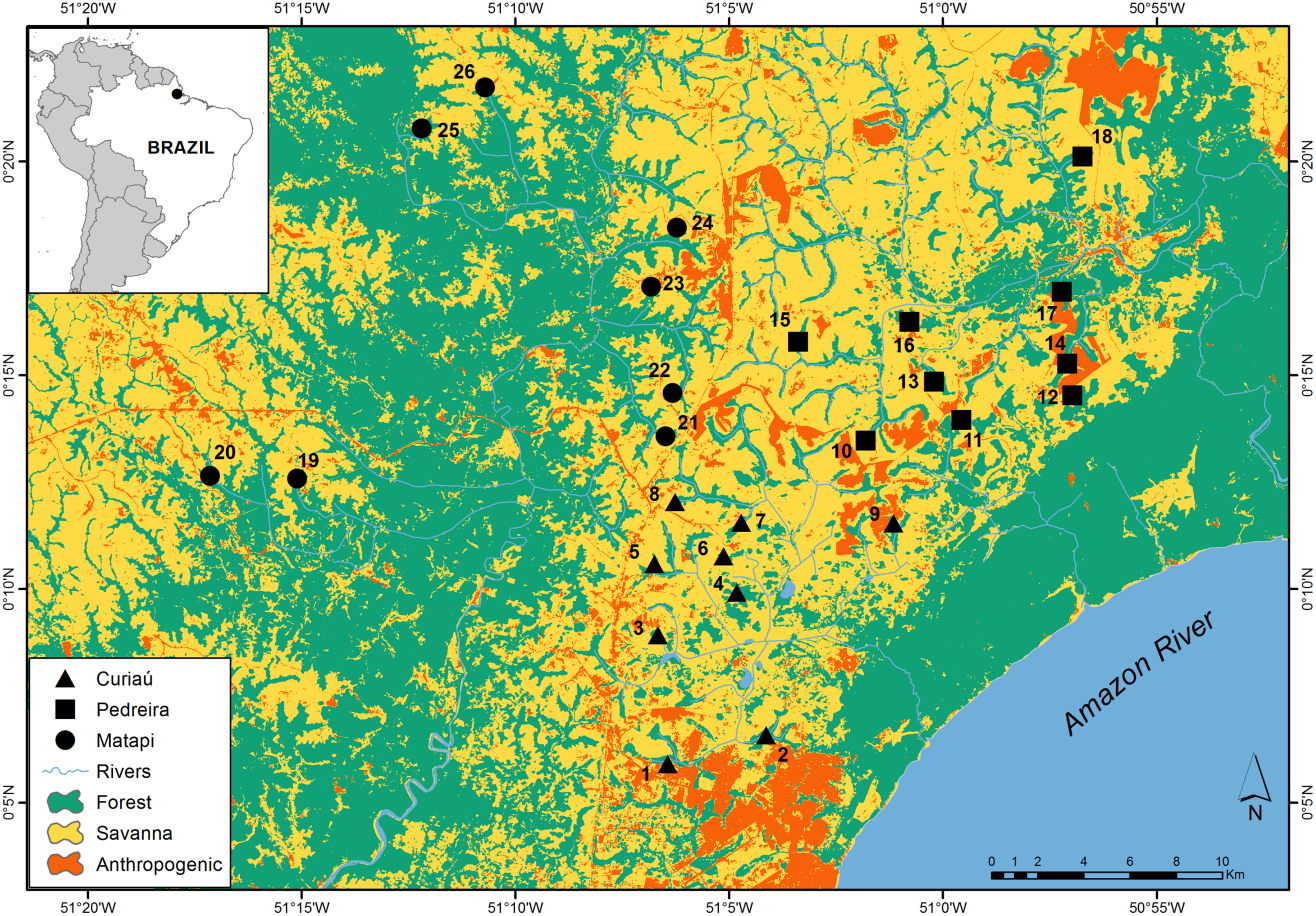
Distribution of the study sites across an Amazonian savanna landscape in Amapá, Brazil. The distribution of the sites by watersheds is as follows: Curiaú (sites 1 to 9), Pedreiras (sites 10 to 18), and Matapi (sites 19 to 16).

We carried out our study in a 123,000-ha savanna landscape (0°12′N, 51°6′W) located between the municipalities of Porto Grande and Macapá, Amapá, Brazil (Fig. 1). This savanna landscape is within the Savannas of Amapá, the third largest block of Amazonian savanna (Carvalho & Mustin, 2017). Currently, this region is considered one of the last agricultural frontiers in Brazil, having been changed mainly due to the cultivation of soybeans, corn, and eucalyptus (Mustin et al., 2017; Hilário et al., 2017). The climate is hot (average temperature of 27 °C) and humid (average relative humidity of 81%) (IEPA, 2008). Average annual precipitation from 1961 to 1990 was ~2,700 mm with a well-marked dry season from August to November, when total monthly rainfall is below 50 mm (Silva et al., 1997).

The study region has three main watersheds: Matapi, Curiaú, and Pedreira (Fig. 1). The rivers and streams of the Matapi watershed connect the study region with the upland tropical forests on Precambrian crystalline terrains located in the west. In contrast, the Curuá and Pedreira watersheds’ streams and rivers connect the study region with the seasonally flooded forests of the Amazon Holocene floodplains located in the east (IEPA, 2008).

The landscape matrix is relatively well conserved. It is dominated by upland savannas and flooded grasslands intersected by gallery forest corridors, with human impacts (mostly small-scale agriculture areas and trails) limited to areas that were once occupied by upland savannas. In general, upland savannas have a ground layer dominated by grass species of the genera *Rhynchospora*, *Axonopu*s, *Paspalum*, *Polygala*, *Bulbostylis*, and *Miconia*, and a woody layer (3–10 m tall) that include large shrubs and trees such as *Byrsonina crassifolia*, *Salvertia convallariodora*, *Ouratea hexasperma*, *Curatella americana*, *Himatanthus articulatus*, *Pallicourea rigida*, and *Hancornia speciosa* (Sanaiotti, Bridgewater & Ratter, 1997). Flooded grasslands are at the bottom of some narrow valleys, where soils are shallow and permanently inundated. These grasslands can sometimes include narrow stands of *Mauritia* and *Mauritiella* palms (Silva et al., 1997). Gallery forests are narrow (80–500 m) and found only on either cambisols or hydromorphic soils rich in organic matter along the wide valleys of rivers and streams (Silva et al., 1997; Costa-Neto, Miranda & Rocha, 2017). They are evergreen with a well-defined canopy composed of 20–30 m tall trees and a humid understory with many ferns, epiphytes, and palms. The most common plant species were *Mauritia flexuosa*, *Euterpe oleracea*, *Mauritiella aculeata*, *Desmoncu*s sp., *Annona paludosa*, *Coccoloba* sp., *Ficus* sp., *Symphonia globulifera*, *Virola* sp., *Lecythis* sp., and *Hymenaea parvifolia* (Costa-Neto, Miranda & Rocha, 2017).

### Site selection

Our study sites were 26 gallery forests associated with low-order streams distributed across the study landscape (Fig. 1). We used two criteria to choose these sites. First, we placed 8–9 sites in each of the three major watersheds in the study region to capture the regional environmental heterogeneity (Fig. 1). Second, we selected the gallery forests that were at least 1.5 km apart within each watershed to enhance spatial independence. In each site, we placed a 500 m long transect to measure habitat structure and count birds, including their abundance and richness. Perpendicularly to these forest transects, we set 400 m linear transects to measure how far species recorded in the gallery forests moved into the savanna. Each transect was marked at 50 m intervals (henceforth, “distance classes”) with colored ribbons nailed to tree trunks.

### Habitat attributes

We quantified habitat attributes of gallery forests by taking measurements of both landscape and vegetation structures. To analyze the landscape structure around each site, we first mapped the landscape’s land cover/use at a spatial resolution of 10 m using Sentinel-2 images (2015–2017) and ArcGis 10.3. Using ArcGIS 10.3, we measured the gallery forest width at the transect centroid as well as estimated the percentage of forests, savannas and anthropogenic ecosystems within 500 m radius around the transect centroid. We used 500 m radius to avoid overlap with the area of influence of other transects.

To measure vegetation structure, we set three plots measuring 75 m × 2 m (150 m^2^) located in the beginning, middle, and end of each transect. In these plots, we took three measurements: (1) canopy cover, (2) understory foliage density, and (3) tree height.

Canopy cover was measured using a leaf coverage index ranging from 0% to 100%. This index is estimated by analyzing the canopy photographs using the program “Gap Light Analysis Mobile App” (GLAMA) (Tichý, 2016). We took four photographs at points located in each plot’s corners, with the camera vertically positioned at the height of 1.6 m above ground. The average leaf coverage of the 12 photographs (four photographs × three plots) was used to measure the site’s canopy cover.

Understory foliage density was measured as the number of foliage contacts between 0.5 and 2 m above ground with an aluminum pole 2 m tall and 5 mm in diameter. The pole was walked in front of the body in the middle of the plot (2 m wide), and all the touches of the three plots were summed and used as a measure of the site’s understory foliage density (Mantovani & Martins, 1990).

Tree height was measured using a 4.5 m graduated ruler and a Bushnell Hypsometer (Scout 1,000 Arc). The laser was focused on the highest branches or leaves, and the hypsometer data was recorded. Then, the formula

$$\rm sin (Angle \times \pi /180) * Distance\ from\ the\ object + 1.59 \; (eye\ height)$$

was used to obtain the height of each tree. We used the site’s average tree height to characterize each site.

### Bird sampling

In the forest transects, birds were counted using 10-min, unlimited radius point counts, one of the most commonly used methods for sampling birds in tropical regions (Bibby et al., 2000; Vielliard et al., 2010). With this method, the observer records all birds seen or heard within a pre-established period. Species flying above the canopy or flying through the sample area were not recorded. A total of 78 points (three points 200-m apart within each transect) was sampled, which was replicated four times. In the savanna transects, birds were counted using the fixed distance transect method, which consists of counting all birds detected visually or aurally within 50 m on each transect side (Bibby et al., 2000).

Because we counted birds twice during the rainy season (April and June 2018) and twice during the dry season (July and September 2018), we are confident that we covered all critical periods of the region’s annual bird cycle. Birds were counted between 06:00 and 10:30, the peak period of bird activity, to maximize detection. The sequence by which transects were sampled was determined randomly one day before the sampling. J.P. surveyed all forest transects while J.C.S sampled all savanna transects. Both surveyors have extensive experience studying birds in the study region.

In each survey, the observer recorded the point location, the start and end time of the count, the recorded species’ identity (observed or heard), and the number of individuals. In the savanna transects, forest’s distance interval was also recorded. In the forest point count, the surveyor tried to avoid double counting vocal species by noting their position in relation to the points. Species flying over the transect were noted but not counted, and therefore were not included in the analysis. Olympus Binoculars (7 × 32) were used in all counts. When necessary to identify a species, vocalizations were recorded with a Zoom H1n recorder and directional microphone Yoga-Ht81.

### Species functional traits

We collected data for each species’ five functional traits included in the analysis: body mass, dispersal ability, diet, foraging stratum, and habitat specificity. These five traits were chosen because previous studies have indicated that they are related to species abundance or range size across different scales, from local to global (*e.g*., Pigot et al., 2018; Valente & Betts, 2019; Spake et al., 2020). Body mass for all species was gathered from the literature (Wilman et al., 2014). We used the Kipp’s Index of wing morphology (KI) as a proxy for dispersal ability because this information is available for all bird species in a standard format (Sheard et al., 2020). Based on field observations and literature (Wilman et al., 2014), we classified species into four dietary groups (nectarivores, herbivores that eat mostly fruits and/or seeds, insectivores that eat primarily insects and other invertebrates, and omnivores that combine herbivore and insectivore diets) and five foraging strata (ground, understory, mid-level, canopy, and edges). As an indicator of a species’ habitat specificity, we used the weighted-average distance in which a forest species was recorded in the different category distances from the forests along the savanna transects.

### Species selection

We gathered 3,770 records from 143 species in all our study sites (Table S1). However, we restricted our analyses to 99 species (representing 2,411 records) associated with forest habitats within savanna landscapes.Thus, we excluded all species considered as forest independent by Silva (1995). Because the methods that we used do not provide a reliable estimate of abundance and occupancy for all groups of birds (Bibby et al., 2000), we also excluded from our analyses all species of Psittacidae (parrots and macaws), Ramphastidae (toucans and toucanets), obligate waterbirds, raptors, nocturnal species, and aerial insectivores.

### Species-level statistical analyses

We estimated abundance and occupancy for each species. Species abundance was estimated at site and at landscape level by dividing the number of points which the species was detected by the total number of points sampled (Hutto, Pletschet & Hendricks, 1986). The occupancy of a species was estimated by dividing the number of sites where the species was present by the total number of sampled sites. We used Pearson’s correlation test to assess the hypotheses that species abundance and species occupancy are associated.

To evaluate if functional traits explain the abundance and occupancy of gallery forest bird species within savanna landscapes, we used Phylogenetic Generalized Least Squares (PGLS) models to avoid problems associated with the statistical nonindependence of related species (Martins & Hansen, 1997). Foraging stratum, a categorical variable, was added to the models using a dummy coding (Mundry, 2014). Phylogenetic distances among species were estimated based on an updated version (available in http://vertlife.org/phylosubsets) of the Jetz et al’s supertree (Jetz et al., 2012) supertree based on the Hackett et al. (2008) bone. Before proceeding with PGLS, we first examined the variance inflation factors (VIF) to ensure that the predictor variables were independent. All variables presented VIF < 3 and were used in the model selection (Dormann et al., 2013). Models were generated and ranked considering Akaike’s information criterion corrected for a small sample size (AICc, Burnham & Anderson, 1998). Models with delta values (Δ_i_) < 2, and high values of Akaike weights (w_i_) (*i.e*., closest to 1), were considered to be those with the most robust support. We computed the best set of models, based on AICc (corrected Akaike Information Criterion), using the “MuMIn” package (Barton, 2020) and the model-averaging procedure. To average models, we computed mean values of estimates assuming (full averages) and not assuming (conditional average) zero values for predictors in models where they did not occur. PGLS model generation and selection were carried out in R using the PGLS function in the package “caper” (Orme et al., 2018). We have followed the recommendation of Revell (2010) and estimated the phylogenetic signal simultaneously using Pagel’s λ (Pagel, 1999) with the regression model.

### Assemblage-level statistical analysis

We measured the three types of alpha diversity: taxonomic, functional, and phylogenetic. To describe alpha diversities, we used the framework described by Chiu & Chao (2014), which is based on Hill numbers. Hill numbers are defined by parameter *q*, which considers the relative abundance of species in determining the estimation of diversity, which facilitates the comparison of data (Hill, 1973; Chiu & Chao, 2014; Roswell, Dushoff & Winfree, 2021). In our case, we only used *q* values that represent taxonomic, functional, and phylogenetic richness (*q* = 0), where the abundance of species is ignored (Hill, 1973; Chiu & Chao, 2014). All Hill numbers were estimated with the R package “hillR” (Li, 2018). For functional richness, the Hill numbers incorporate an array of functional distances constructed from the functional traits of the species (see Chiu & Chao, 2014). For phylogenetic richness, the Hill numbers incorporate a phylogenetic tree (Li, 2018).

We used hierarchical partitioning (Chevan & Sutherland, 1991; Cisneros, Fagan & Willig, 2015) to identify the habitat attributes that best accounted for variation in each of the three dimensions of biodiversity. Statistical significance of the independent contribution of each explanatory variable was determined using a randomization approach with 1,000 iterations and an alfa-level of 0.05 (MacNaly & Walsh, 2020). Hierarchical partitioning and associated randomization tests were executed using the R package ‘hier.part’ (MacNaly & Walsh, 2020).

To test the hypothesis that less diverse bird assemblages are a nested subset of more diverse assemblages, we carried out taxonomic, phylogenetic, and functional nestedness analyses. Presence-absence matrices were first constructed where species were in the columns and sites were in the rows. Taxonomic nestedness was then estimated using the NODF index (Nestedness Metric Based on Overlap and Decreasing Fill). We chose NODF because it has more robust statistical properties than other indices and quantifies the degree to which each site is nested in each of the other sites (Almeida-Neto et al., 2008). We evaluated the significance of the taxonomic nestedness using the fixed–fixed null model (999 permutations) based on the “*quasiswap”* algorithm (Miklós & Podani, 2004). Both NODF estimation and the significance test were conducted using the R package ‘vegan’ (Oksanen et al., 2019).

To estimate functional (traitNODF) and phylogenetic (phyloNODF) nestedness, we used an extension of the NODF index called treeNODF index (Melo, Cianciaruso & Almeida-Neto, 2014), the same phylogeny used for the Phylogenetic Generalized Least Squares (PGLS) models, and a functional dendrogram created by using five functional traits (body mass, wing morphology, dispersal potential, diet, and foraging stratum). The functional dendrogram represents species similarity for the five functional traits and was generated from the function *gawdis* and UPGMA clustering algorithm. We used the *gawdis* function because there are problems in combining quantitative and categorical traits into multi-trait dissimilarities using Gower distance (Pavoine et al., 2009). Function *gawdis* balances the different traits when computing multi-trait dissimilarities, finding weights that minimize the differences in the correlation between the dissimilarity of each trait and the multi-trait (Bello et al., 2020). In general, the treeNODF index assesses the proportion of functional/phylogenetic diversity present in functionally/phylogenetically impoverished assemblages that are present in functionally/phylogenetically rich assemblages (Melo, Cianciaruso & Almeida-Neto, 2014). In addition, we partitioned the traitNODF and phyloNODF into their two components: S.fraction and topoNODF. The S.fraction represents the degree to which assemblages are or are not nested due to having assemblages composed of the same or different species. In contrast, topoNODF represents the degree to which assemblages are nested or not within the functional dendrogram or phylogenetic tree (Melo, Cianciaruso & Almeida-Neto, 2014). The treeNODF index was estimated using the R package ‘CommEcol’ (Melo, 2019). The significance of the observed traitNODF and phyloNODF and their component values (S.fraction and topoNODE) were determined using a permutation null model (999 permutations).

## Results

### Species abundance and occupancy

Species abundance and species occupancy are positively correlated (Pearson’s correlation coefficient, r = 0.91, df = 97, *p* < 0.001). Among the 99 species included in our analyses (Table S1, Fig. S1), most of them have a low abundance index (range = 0.95–125.9, median = 12.5) and low occupancy index (range = 0.038–1, median = 0.26). Four species had the highest abundance indices (Fig. S1). Three of them were recorded in all 26 sites: a hummingbird (*Phaethornis ruber*), a small insectivore flycatcher (*Lophotriccus galeatus*), and an omnivorous thrush (*Turdus leucomelas*). However, the most abundant species (a small insectivore flycatcher, *Tolmomyias flaviventris*) was recorded in 25 sites. Among the rarest species, 26 species were recorded in less than two sites (Fig. S1). All of them had low abundance indices (range = 0.96–2.88, median = 1.92). Most of the rare species are interior forest species, including, for instance, a large tinamou (*Tinamus major*), six woodcreepers (*Dendrexetastes rufigula*, *Dendrocolaptes certhia*, *Dendrocolaptes picumnus*, *Lepidocolaptes albolineatus*, *Nasica longirostris and Xiphorhynchus obsoletus*) and two antbirds (*Myrmoderus ferrugineus*, *Mymophylax atrothorax*).

**Figure 2:**
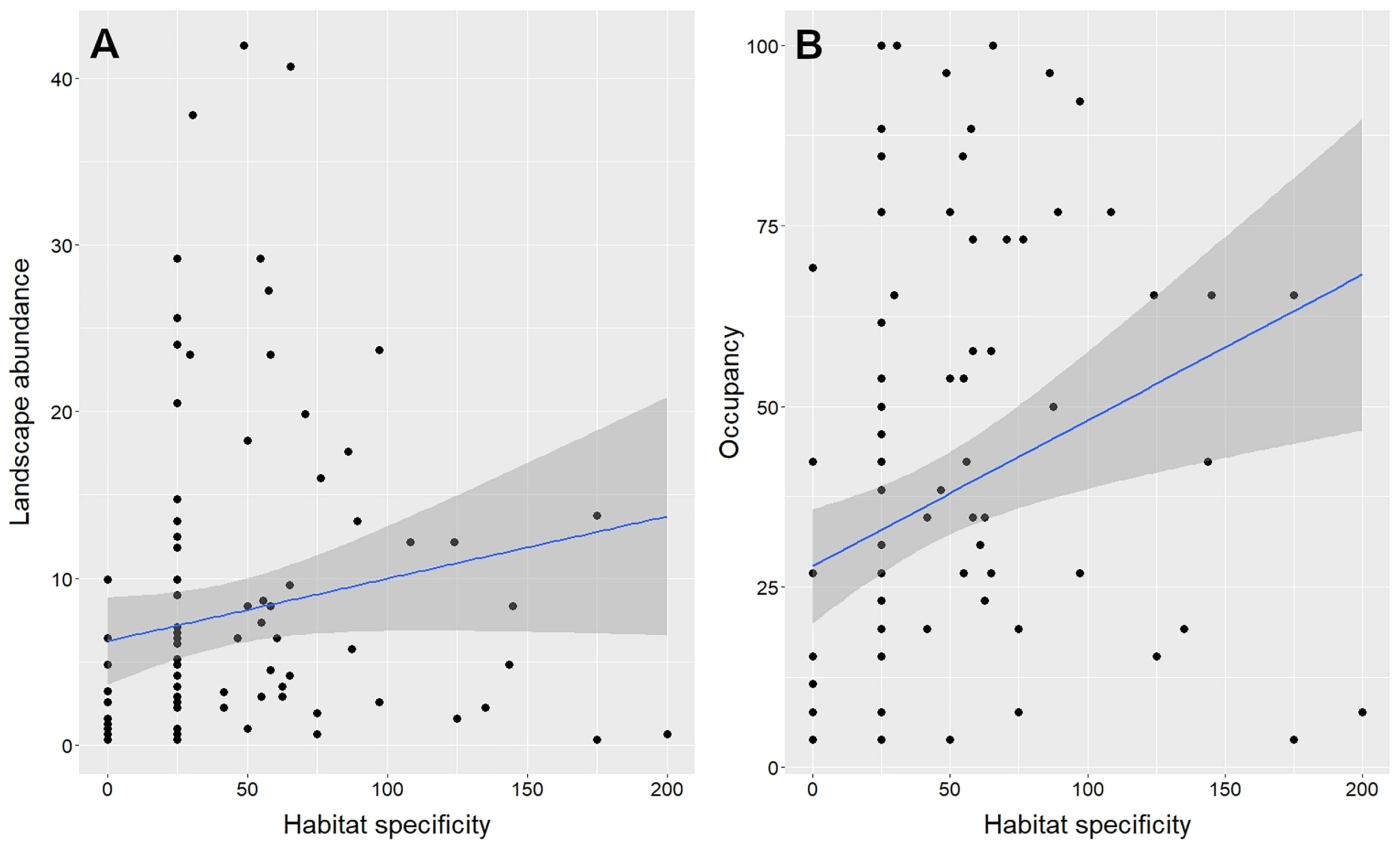
Univariate relationship between habitat specificity and landscape abundance (A) and occupancy (B) of gallery forest birds in an Amazonian savanna landscape, Amapá, Brazil. Each data point represents one of the 99 species evaluated. The shaded areas represent the 95% confidence intervals.

The best models (*i.e*., the ones with the lowest AICc) predicting species abundance (Table S2) and species occupancy (Table S3) from functional traits included four out of five functional traits examined: habitat specificity, Kipp’s Index, foraging stratum groups, and body mass. However, habitat specificity is the only one of these traits that has a positive and significant correlation with both abundance and occupancy (Table 1, Fig. 2).

**Table 1:** Model-averaged parameter estimates (standard deviation in parentheses) of phylogenetic generalized least squares models relating species traits, abundance and occupancy of 99 gallery forest birds in an Amazonian savanna landscape, Amapá, Brazil.

	Mean landscape abundance			Occupancy		
	Estimate	z value	*p* value	Estimate	z value	*p* value
(Intercept)	4.673	0.624	0.532	23.307	0.941	0.346
Habitat specificity	0.065	2.321	0.020	0.263	3.475	<0.001
Foraging stratum–Ground	−2.899	0.621	0.534	−16.362	0.924	0.355
Foraging stratum–Mid-level	−0.160	0.061	0.951	−3.696	0.380	0.703
Foraging stratum–Canopy and Edge	−3.047	0.866	0.386	−4.583	0.467	0.640
Body mass	1.028	0.447	0.654	5.099	0.602	0.546
Dispersal ability	0.034	0.339	0.734	0.082	0.283	0.777

### Species assemblages

**Figure 3:**
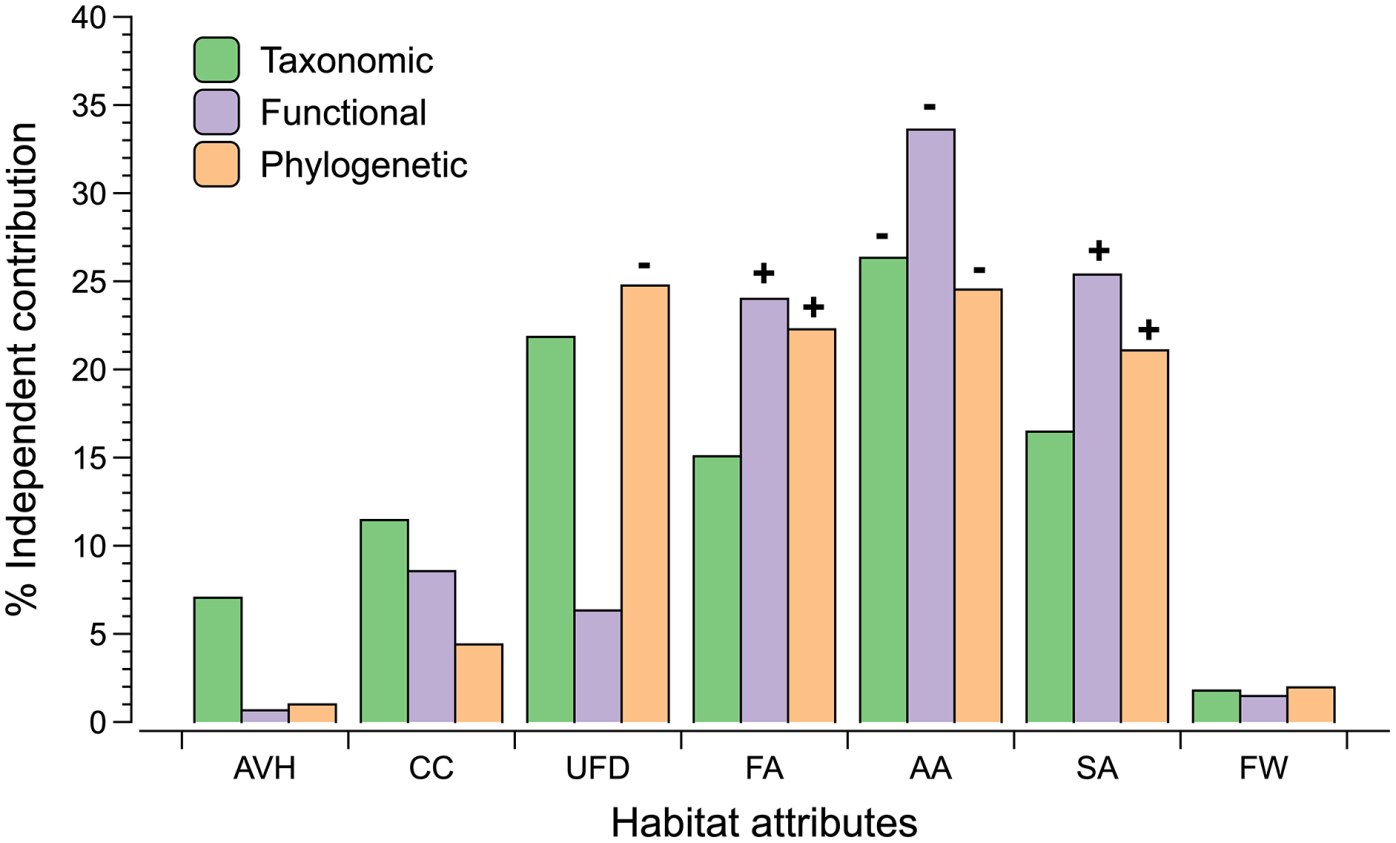
The percentage independent contribution of each habitat attributes (vegetation or landscape) derived by hierarchical partitioning on each dimension of biodiversity. Codes for habitat attributes are AVH (Average height vegetation), CC (Canopy cover), UFD (Understory foliage density), FA (percentage of forest cover within 500 m buffer), AA (percentage of anthropogenic cover within 500 m buffer), SA (percentage of savanna cover within 500 m buffer), and FW (forest width). Significant results (*P* ≤ 0.05) are indicated with a positive or negative sign that indicates the direction of the correlation between the dimension of biodiversity and the habitat attribute.

We detected different patterns of relationships between indicators of landscape and vegetation structure with diversity estimates (Fig. 3; Table S4). The proportion of anthropogenic area around the sites was negatively correlated with taxonomic, functional and phylogenetic diversity. In contrast, forest and savanna areas are associated positively with functional and phylogenetic diversity. Understory foliage density is negatively associated with phylogenetic diversity.

**Figure 4:**
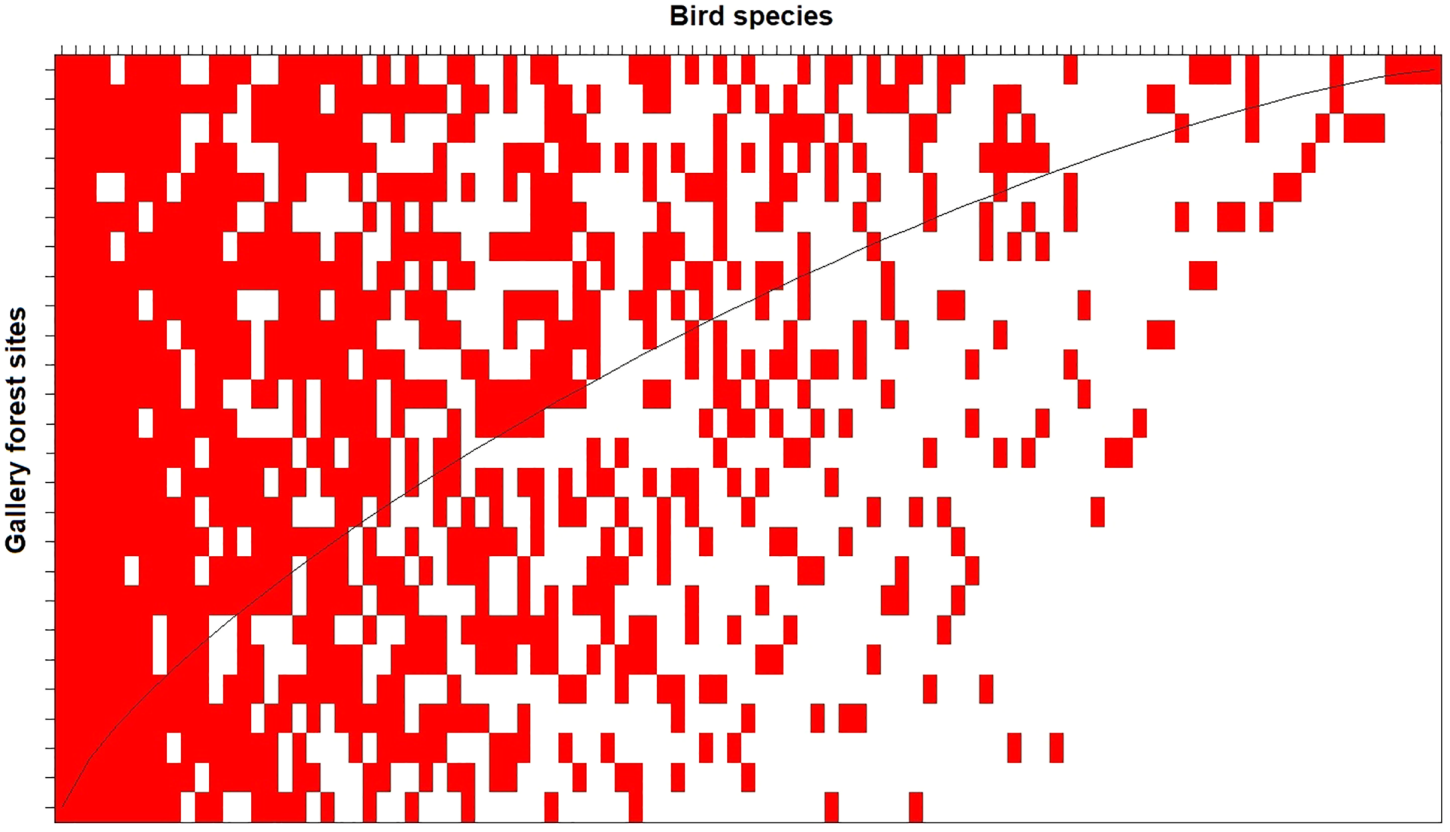
Taxonomic nestedness degree of the 26 local gallery forest bird assemblages in an Amazonian savanna landscape, Amapá, Brazil. The columns represent the species, and the lines represent the sampled sites. The sites (rows) would be perfectly nested if all interactions were above the “fill line” (black curved line).

The nestedness analysis indicated that the less diverse assemblages are nested subsets of the most diverse assemblages (Fig. 4). This pattern holds when analyzing taxonomic (NODF = 53.5, *p* < 0.01; Figure 4), phylogenetic (phyloNODF = 64.6, *p* < 0.01) and functional (treeNODF = 67.1, *p* < 0.01) nestedness. In addition, we found that phylogenetic and functional nestedness is driven mostly by changes in taxonomic species composition (S.fraction = 52.9 and 54.6, respectively) rather than by functional or phylogenetic tree topology (topoNODF = 14.1 and 10.1, respectively).

## Discussion

Species abundance and species occupancy of gallery forest birds are correlated at the landscape level. This result matches what has been reported in several studies at multiple spatial and temporal scales (Gaston et al., 2000; Borregaard & Rahbek, 2010; Webb, Freckleton & Gaston, 2012). This general pattern is possibly an outcome of the interactions between resource-based and population dynamic mechanisms (Gaston, 2003; Borregaard & Rahbek, 2010). Resource-based mechanisms are determined by habitat attributes, which set the spatial distribution and size of potentially habitable areas of a species in a landscape. In contrast, population dynamic mechanisms, such as population growth, habitat specificity, and dispersal ability, are determined by species functional traits and set a proportion of the habitable sites that a species occupies at any given time. Our results indicate that only habitat specificity can be considered as a robust predictor of both species abundance and occupancy in gallery forests among all functional traits examined. Among gallery forest birds, those that move deeper into the savanna matrix for at least part of their annual life cycles are the ones most likely to maintain large local populations and occupy more gallery forests.

In general, species assemblages living in gallery forests are dominated by generalist species that can use savannas to spread out across the landscape but not by species with high dispersal capacity. Therefore, our results only partially support Forman’s hypothesis that most species living in these habitats should exhibit both traits (Forman, 1995). In addition, we found that gallery forests can also maintain populations of forest interior species although in low population density. For instance, if we consider all species foraging in the midstory, understory, and ground, more than half of the species we recorded in our sites can be classified as forest interior species. Dominant species in gallery forests are either forest canopy or early-successional species that are more tolerant to habitat changes and open spaces. Within the Amazon, they are found mostly in seasonally flooded forests and second-growth forests (Novaes, 1973; Silva, Uhl & Murray, 1996; Borges, 2007) and are rare or absent in landscapes dominated by pristine continuous forests (Rutt et al., 2019). Although versatile in their habitat preferences, these species are not core components of savanna bird assemblages (Boss & Silva, 2015) and their presence within savanna landscapes requires gallery forests (Silva, 1995). Gallery forest avifaunas inhabiting Amazonian savannas are not novel assemblages formed by the influence of human activities in the region (Young, 2014). In contrast, they are unique assemblages composed of species derived from the different forest ecosystems that surround the landscapes dominated by savanna vegetation.

Indicators of landscape structure explained more of the variation of the three biodiversity dimensions than indicators of vegetation structure. Among the indicators of vegetation structure only understory foliage density was negatively correlated with phylogenetic diversity, indicating that foliage density can have negative effects on the abundance of some monophyletic groups of birds that live in the forest interior and were represented in our sample. We found that increasing human activities in the landscape is negatively correlated with taxonomic, functional and phylogenetic diversity of gallery forests. Furthermore, our results show that the proportion of savanna and forest around the sites is positively associated with functional and phylogenetic diversity. This is a new finding and supports the notion that in tropical landscapes, extrinsic factors, such as matrix dynamics, are at least as important as intrinsic factors to explain the ecological processes operating within habitat patches (Gascon et al., 1999; Boesing, Nichols & Metzger, 2018; Stouffer, 2020). Because gallery forests are important to the maintenance of freshwater resources relevant to human activities, human pressures on neotropical savanna landscapes occur generally on the upland savannas adjacent to gallery forests, rather than in the gallery forest themselves (Mustin et al., 2017). We suggest that land cover changes in the savanna matrix negatively influence the taxonomic, functional and phylogenetic diversity of bird assemblages of gallery forests by simultaneously increasing isolation and edge effects. In tropical forests, both isolation and edge effects are known to reduce the diversity of neotropical forest birds (Lees & Peres, 2006, 2009; Banks-Leite, Ewers & Metzger, 2010; Stouffer, 2020). Isolation reduces species diversity by reducing the flow of individuals and species between habitat patches and thus increasing the likelihood of random local extinction. Gallery forests birds can move across the landscape either by following the gallery forest networks along the rivers or by crossing the savanna matrix. If birds that cross the savanna matrix are not able to use the anthropogenic vegetation that surround them, then they can become partially isolated and, over time, decline (Tubelis, Cowling & Donnelly, 2004; Tubelis, Lindenmayer & Cowling, 2004). On the other hand, edge effects reduce species diversity by eliminating microhabitats used by specialist forests species. Although edge effects are the norm in gallery forests because they are naturally narrow habitats (de Oliveira Coelho et al., 2016), they tend to increase substantially if the adjacent upland savannas are removed and replaced by agriculture fields (Nóbrega et al., 2020).

As predicted, gallery forest bird species are not randomly distributed across tropical savanna landscapes. Instead, less diverse bird assemblages are a nested subset of more diverse assemblages generally located near the areas of continuous forests. This nestedness pattern holds when considering all three dimensions (taxonomic, functional, and phylogenetic) of species diversity. Moreover, we found that nestedness is driven mostly by changes in species composition across sites. Several biological processes can explain nestedness in biological assemblages, but the most likely alternatives are selective extinction and selective colonization (Ulrich, Almeida-Neto & Gotelli, 2009). Nestedness by selective extinction occurs when a habitat is retracting in a region. As a consequence, species are locally extinct because they have different susceptibilities to habitat fragmentation and reduction (Patterson, 1987, 1990). On the other hand, selective colonization occurs when the habitat in a region is expanding from a place with a species pool composed of species with different dispersal abilities (Darlington, 1957; Kadmon, 1995; Dardanelli & Bellis, 2020). Because gallery forests are expanding rather than retracting over neotropical savannas under the current climate (Cole, 1986; Oliveira-Filho & Ratter, 1995), we suggest that selective colonization is the most likely process leading to nestedness in gallery forest bird assemblages. However, our results show that selective colonization can be mediated by habitat filtering, as forest species with low habitat specificity can use forest expansion to colonize more gallery forests patches than species with high habitat specificity. This inter-specific difference in habitat specificity results in a pattern in which due to taxonomic turnover across sites, the most diverse gallery forests support species with both high and low habitat specificity, whereas less diverse gallery forests support only the ones with low habitat specificity.

Three main recommendations for the long-term management of gallery forests can be proposed based on our results. First, maintaining the connectivity between gallery forests and adjacent continuous forests is essential because gallery forest bird assemblages are derived from continuous forest species assemblages through a process of selective colonization. Second, because most species use the savanna matrix to move across the landscape, effectively managing the savanna matrices where gallery forests are embedded is as important to maintaining viable populations of forest bird species as managing the gallery forest themselves. Third, in savanna landscapes planned to be used for agriculture production, protecting gallery forests alone is not enough. Instead, gallery forests should be protected with surrounding savanna buffers to avoid the detrimental effects (edge effects and isolation) of human activities on their biodiversity. Although several countries have specific legislation to safeguard the connectivity of gallery forests due to their importance for water protection and flood regulation, they usually do not consider the importance of managing the savanna matrix or the maintenance of savanna buffers (Tubelis, Cowling & Donnelly, 2004). In Brazil, for instance, there is a modern conservation law that regulates the use of native ecosystems on private lands (Law N°.12,651/12). This law considers gallery forests to be APPs (Áreas de Proteção Permanente or Permanent Preservation Areas), which are areas set aside to forever preserve water resources, stability (of the landscape, soil, and geology), biodiversity (facilitating the gene flow of fauna and flora), and human well-being (Silva, Pinto & Scarano, 2021). The law defines parameters (*e.g*., river widths, slopes, and altitude) for landowners to demarcate these APPs, but the management of the savanna matrix and the inclusion of savanna conservation buffers around gallery forests are not included among these parameters.

## Conclusion

Our results show that gallery forests are important biodiversity reservoirs in savanna landscapes because they maintain populations of both forest dependent and semi-dependent species that are not able to live in savannas (Silva, 1996; Silva et al., 1997; Piratelli & Blake, 2006). In addition, we found that: (1) habitat specificity is the only functional trait that predicts species abundance and occupancy across a landscape; (2) phylogenetic diversity is negatively correlated with understory foliage density; (3) the percentage of forests and savannas around sites is positively correlated with both phylogenetic and functional diversity; (4) increasing human activities around gallery forest negatively influences taxonomic and functional diversity; and (5) forest bird assemblages are not distributed at random across the landscape but show a nested pattern caused by selective colonization mediated by habitat filtering. Altogether, these findings provide a more nuanced perspective on how forest birds are distributed in a tropical savanna landscape and guidance for the designing of sound conservation strategies for gallery forest bird assemblages.

## Supplemental Information

10.7717/peerj.12529/supp-1 Supplemental Information 1 Raw Vegetation Data.

10.7717/peerj.12529/supp-2 Supplemental Information 2 Forest bird data counts.

10.7717/peerj.12529/supp-3 Supplemental Information 3 Savanna bird data count.

10.7717/peerj.12529/supp-4 Supplemental Information 4 Figure S1 and Tables S1-S4.

## Additional Information and Declarations

### Field Study Permissions

The following information was supplied relating to field study approvals (*i.e*., approving body and any reference numbers): The study was conducted in private lands. No formal request was required.

### Data Availability

The following information was supplied regarding data availability: All raw measurements (bird counts and habitat structure) are available in the Supplementary Files.
