# Supplementary material for: Composition, richness and nestedness of gallery forest bird assemblages in an Amazonian savanna landscape: lessons for conservation
Source: PeerJ. 2021 Dec 1;9:e12529. doi: 10.7717/peerj.12529 (PMC8643102; doi:10.7717/peerj.12529)
Supplement: Supplemental Information 4 [file peerj-09-12529-s004.pdf]

Supplemental material

Composition, richness and nestedness of gallery forest bird assemblages in an Amazonian savanna landscape: lessons for conservation

Joandro Pandilha[1], José Júlio Toledo[1], Luis Claudio Fernandes Barbosa[2], William Douglas de Carvalho[3], Jackson Cleiton de Sousa[1] & José Maria Cardoso da Silva[4]

[1] Universidade Federal do Amapá, Programa de Pós-Graduação em Biodiversidade Tropical, Campus Universitário Marco Zero do Equador, Rodovia Juscelino Kubitschek, km 02, Bloco T, Bairro Universidade, 68903-419, Macapá, AP, Brazil

[2] Conservação Internacional do Brasil, 14 de Abril 1186, Apt 205, 66060-460 Belém, Pará, Brazil.

[3] Universidade Federal da Grande Dourados, Programa de Pós-Graduação em Biodiversidade e Meio Ambiente, Rodovia Dourados/Itahum,, Km12, 79804-970, Dourados, MS, Brazil.

[4] University of Miami, Department of Geography and Sustainable Development, 1300 Campo Sano, Coral Gables, FL, 33124-4401

Contents:

Figure S1

Tables S1-S4

Figure S1: Mean landscape abundance and occupancy of 99 bird species recorded in 26 gallery forests of an Amazonian savanna landscape, Amapá, Brazil.

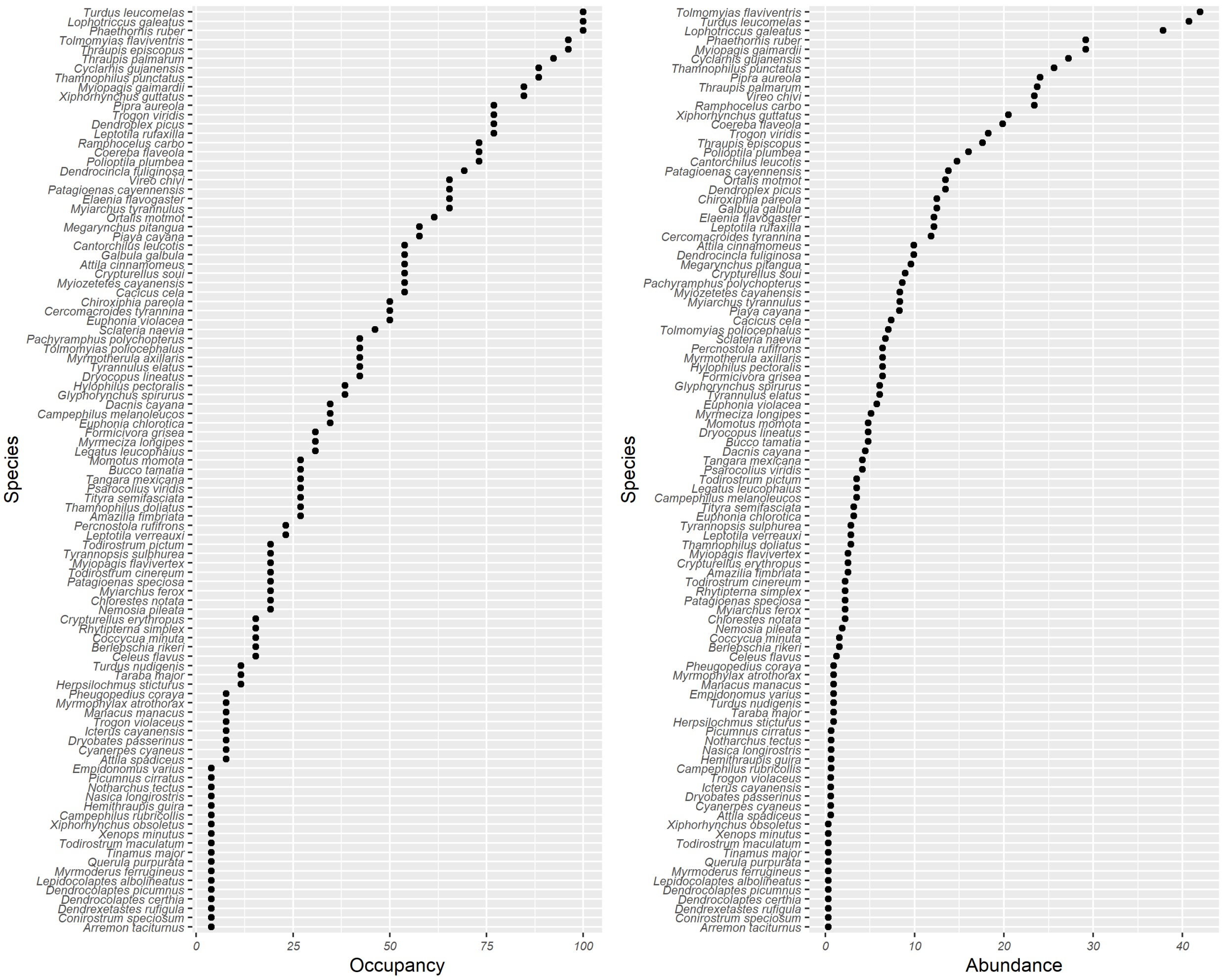

Table S1: Bird species recorded in 26 gallery forests of an Amazonian savanna landscape, Amapá, Brazil, and their respective functional traits. Species that do not have information about functional traits were not well sampled in our surveys and excluded from all statistical analyses.

Order/Family/Species	Landscape mean abundance	Occupancy	Body mass (log-transformed)	Trophic groups	Wing morphology	Dispersal ability	Foraging stratum groups
Tinamiformes							
Tinamidae							
Tinamus major	0.32	3.8	3.05	omnivore	20.50	0.0	ground
Crypturellus soui	8.97	53.8	2.31	omnivore	26.76	25.0	Ground
Crypturellus erythropus	2.56	15.4	2.54	omnivore	30.14	0.0	Ground
Galliformes							
Cracidae							
Ortalis motmot	13.46	61.5	2.70	frugivore-seeds	14.84	25.0	canopy and edges
Columbiformes							
Columbidae							
Leptotila verreauxi	2.88	23.1	2.19	frugivore-seeds	21.85	62.5	ground
Leptotila rufaxilla	12.17	76.9	2.19	frugivore-seeds	24.39	108.3	canopy and edges
Patagioenas cayennensis	13.77	65.4	2.41	frugivore-seeds	34.06	175.0	canopy and edges
Patagioenas speciosa	2.44	19.2	2.42	frugivore-seeds	36.51	25.0	canopy and edges
Cuculiformes							
Cuculidae							
Coccycua minuta	1.60	15.4	1.48	insectivore	14.61	125.0	canopy and edges
Piaya cayana	8.33	57.7	2.07	insectivore	11.30	58.3	canopy and edges
Caprimulgiformes							
Trochilidae							
Phaethornis ruber	29.17	100	0.34	nectarivore	74.80	25.0	understory

Amazilia fimbriata	2.56	26.9	0.63	nectarivore	63.00	97.2	mid-level
Chlorestes notata	2.24	19.2	0.60	nectarivore	67.02	25.0	mid-level
Trogoniformes							
Trogonidae							
Trogon viridis	18.27	76.9	1.92	omnivore	33.19	50.0	mid-level
Trogon violaceus	0.64	7.7	1.68	omnivore	38.40	25.0	mid-level
Coraciiformes							
Momotidae							
Momotus momota	4.81	26.9	2.09	insectivore	18.58	0.0	canopy and edges
Galbuliformes							
Bucconidae							
Notharchus tectus	0.64	3.8	1.50	insectivore	27.19	25.0	mid-level
Bucco tamatia	4.80	26.9	1.47	insectivore	22.39	25.0	mid-level
Galbuliformes							
Galbulidae							
Galbula galbula	12.5	53.8	1.46	insectivore	24.60	25.0	mid-level
Piciformes							
Ramphastidae							
Pteroglossus aracari	-	-	-	-	-	-	-
Ramphastos tucanus	-	-	-	-	-	-	-
Ramphastos vitellinus	-	-	-	-	-	-	-
Picidae							
Picumnus cirratus	0.64	3.8	1.00	insectivore	11.55	25.0	mid-level
Dryobates passerinus	0.64	7.7	1.48	insectivore	15.35	200.0	mid-level
Campephilus rubricollis	0.64	3.8	2.34	insectivore	17.73	0.0	mid-level
Campephilus melanoleucos	3.52	34.6	2.36	insectivore	22.08	62.5	canopy and edges
Dryocopus lineatus	4.80	42.3	2.31	insectivore	20.46	143.8	canopy and edges
Celeus flavus	1.28	15.4	2.30	insectivore	19.30	0.0	canopy and edges

Psittaciformes							
Psittacidae							
Brotogeris sanctithomae	-	-	-	-	-	-	-
Brotogeris versicolurus	-	-	-	-	-	-	-
Graydidascalus brachyurus	-	-	-	-	-	-	-
Amazona ochrocephala	-	-	-	-	-	-	-
Amazona amazonica	-	-	-	-	-	-	-
Pyrrhura picta	-	-	-	-	-	-	-
Orthopsittaca manilatus	-	-	-	-	-	-	-
Ara ararauna	-	-	-	-	-	-	-
Ara macao	-	-	-	-	-	-	-
Ara severus	-	-	-	-	-	-	-
Psittacara leucophthalmus	-	-	-	-	-	-	-
Passeriformes							
Thamnophilidae							
Taraba major	0.92	11.5	1.78	insectivore	5.18	0.0	understory
Thamnophilus doliatus	2.88	26.9	1.45	insectivore	8.99	55.0	understory
Thamnophilus punctatus	25.64	88.5	1.29	insectivore	10.06	25.0	understory
Myrmotherula axillaris	6.41	42.3	0.92	insectivore	15.21	0.0	understory
Herpsilochmus sticturus	0.96	11.5	1.00	insectivore	15.40	0.0	mid-level
Formicivora grisea	6.40	30.8	1.03	insectivore	8.15	60.7	canopy and edges
Cercomacroides tyrannina	11.85	50. 0	1.26	insectivore	9.29	25.0	understory
Sclateria naevia	6.73	46.2	1.34	insectivore	12.75	25.0	understory
Percnostola rufifrons	6.41	23.1	1.46	insectivore	15.01	25.0	Understory
Myrmeciza longipes	5.13	30.8	1.34	insectivore	6.40	25.0	understory
Myrmoderus ferrugineus	0.32	3.8	1.39	insectivore	6.62	0.0	ground
Myrmophylax atrothorax	0.96	7.7	0.90	insectivore	13.43	0.0	ground
Furnariidae							

Dendrocincla fuliginosa	9.92	69.2	1.57	insectivore	18.33	0. 0	mid-level
Glyphorynchus spirurus	6. 09	38.5	1.13	insectivore	23.92	25.0	mid-level
Dendrexetastes rufigula	0.32	3.8	1.85	insectivore	14.68	0.0	mid-level
Nasica longirostris	0.64	3.8	1.96	insectivore	18.75	0.0	mid-level
Dendrocolaptes certhia	0.32	3.8	1.83	insectivore	17.81	0.0	mid-level
Dendrocolaptes picumnus	0.32	3.8	1.89	insectivore	17.03	0.0	mid-level
Xiphorhynchus obsoletus	0.32	3.8	1.59	insectivore	18.68	0.0	mid-level
Xiphorhynchus guttatus	20.51	84.6	1.81	insectivore	16.16	25.0	mid-level
Dendroplex picus	13.45	76.9	1.61	insectivore	17.80	89.3	mid-level
Lepidocolaptes albolineatus	0.32	3.8	1.43	insectivore	21.72	0.0	canopy and edges
Xenops minutus	0.32	3.8	1.08	insectivore	21.97	0.0	mid-level
Berlepschia rikeri	1.60	15.4	1.48	insectivore	31.73	0.0	canopy and edges
Pipridae							
Chiroxiphia pareola	12.51	50.0	1.29	frugivore-seeds	10.37	25.0	understory
Manacus manacus	0.96	7.7	1.06	frugivore-seeds	15.70	25.0	canopy and edges
Pipra aureola	24. 03	76.9	1.18	frugivore-seeds	19.31	25.0	Understory
Cotingidae							
Querula purpurata	0.32	3.8	2.10	omnivore	17.98	0.0	mid-level
Tityridae							
Tityra semifasciata	3.20	26.9	1.94	omnivore	25.71	0.0	canopy and edges
Pachyramphus polychopterus	8.65	42.3	1.31	omnivore	20.82	55.8	canopy and edges
Tyrannidae							
Lophotriccus galeatus	37.82	100.0	1.00	insectivore	17.25	30.6	mid-level
Todirostrum maculatum	0.32	3.8	0.88	insectivore	13.82	25.0	canopy and edges
Todirostrum cinereum	2.24	19.2	0.85	insectivore	16.65	41.7	canopy and edges
Todirostrum pictum	3.52	19.2	0.85	insectivore	15.14	25.0	canopy and edges
Tolmomyias poliocephalus	7. 05	42.3	1.04	insectivore	14.86	25.0	canopy and edges
Tolmomyias flaviventris	41.98	96.2	1.15	insectivore	16.21	48.7	canopy and edges

Tyrannulus elatus	6. 09	42.3	0.86	insectivore	19.10	25.0	canopy and edges
Myiopagis gaimardii	29.17	84.6	1.10	insectivore	18.36	54.5	mid-level
Myiopagis flavivertex	2.56	19.2	1.04	insectivore	17.44	25.0	canopy and edges
Elaenia flavogaster	12.18	64.4	1.30	omnivore	14.91	124.0	canopy and edges
Attila cinnamomeus	9.93	53.8	1.52	omnivore	9.04	25.0	mid-level
Attila spadiceus	0.64	7.7	1.54	omnivore	14.75	75.0	mid-level
Rhytipterna simplex	2.24	15.4	1.53	insectivore	16.73	25.0	mid-level
Myiarchus ferox	2.24	19.2	1.38	omnivore	11.98	135.0	canopy and edges
Myiarchus tyrannulus	8.33	65.4	1.42	insectivore	14.06	145.0	canopy and edges
Megarynchus pitangua	9.61	57.7	1.78	insectivore	22.67	65.0	canopy and edges
Myiozetetes cayanensis	8.33	53.8	1.62	insectivore	15.42	50.0	canopy and edges
Legatus leucophaius	3.52	30.8	1.39	frugivore-seeds	25.44	25.0	canopy and edges
Empidonomus varius	0.96	3.8	1.42	Insectivore	20.82	50.0	canopy and edges
Tyrannopsis sulphurea	2.88	19.2	1.48	insectivore	16.27	25.00	mid-level
Vireonidae							
Cyclarhis gujanensis	27.24	88.5	1.45	omnivore	17.93	57.7	canopy and edges
Hylophilus pectoralis	6.41	38.5	1.04	insectivore	13.86	46.4	canopy and edges
Vireo chivi	23.40	65.4	1.18	omnivore	24.08	29.5	canopy and edges
Polioptilidae							
Polioptila plumbea	16. 0	73.1	0.78	insectivore	12.33	76.4	canopy and edges
Troglodytidae							
Pheugopedius coraya	0.96	7.7	1.22	insectivore	11.56	0.0	Understory
Cantorchilus leucotis	14.75	53.8	1.20	insectivore	12.18	25.0	understory
Turdidae							
Turdus leucomelas	40.71	100.0	1.83	omnivore	22.52	65.6	canopy and edges
Turdus nudigenis	0.96	11.5	1.81	omnivore	20.01	0.0	canopy and edges
Fringillidae							
Euphonia chlorotica	3.20	34.6	0.90	frugivore-seeds	23.22	41.7	canopy and edges

Euphonia violacea	5.76	50.0	1.15	frugivore-seeds	28.07	87.5	canopy and edges
Passerellidae							
Arremon taciturnus	0.32	3.8	1.45	omnivore	10.87	0.0	Ground
Icteridae							
Psarocolius viridis	4.17	26.9	2.48	insectivore	23.43	25.0	canopy and edges
Cacicus cela	7.37	53.8	1.93	omnivore	27.53	55.0	canopy and edges
Icterus cayanensis	0.64	7.7	1.62	insectivore	15.45	25.0	canopy and edges
Thraupidae							
Nemosia pileata	1.92	19.2	1.15	insectivore	20.17	75.0	canopy and edges
Ramphocelus carbo	23.40	73.1	1.39	omnivore	17.36	58.3	canopy and edges
Thraupis episcopus	17.63	96.2	1.54	omnivore	22.94	86.1	canopy and edges
Thraupis palmarum	23.72	92.3	1.59	omnivore	21.07	97.2	canopy and edges
Tangara mexicana	4.17	26.9	1.28	omnivore	22.06	65.0	canopy and edges
Dacnis cayana	4.48	34.6	1.11	omnivore	24.41	58.3	canopy and edges
Cyanerpes cyaneus	0.64	7.7	1.14	frugivore-seeds	27.05	0.0	canopy and edges
Hemithraupis guira	0.64	3.8	1.00	insectivore	22.56	0.0	canopy and edges
Conirostrum speciosum	0.32	3.8	0.95	insectivore	22.80	175.0	canopy and edges
Coereba flaveola	19.88	73.1	0.97	nectarivore	19.30	70.6	canopy and edges

Table S2: Results of model selection from phylogenetic generalized least squares models relating species functional traits with the landscape mean abundance of 99 bird species recorded in 26 gallery forests of an Amazonian savanna landscape, Amapá, Brazil. Models are ranked according to their AICc. Sample-size adjusted AIC (AICc), Akaike differences (Δi) and Akaike weights (wi) are given

Model	Intercept	Wing morphology	Canopy and Edge	Ground	Habitat specificity	Body mass	Midstory	df	logLik	AIC_c	Δ_i	w_i
11	6.801		-3.866		0.072			3	-369.512	745.3	0	0.094
15	8.792		-4.581	-6.534	0.068			4	-368.519	745.5	0.19	0.085
9	5.608				0.063			2	-370.858	745.8	0.56	0.071
31	3.274		-4.908	-6.924	0.070	3.174		5	-367.936	746.5	1.24	0.05
27	1.778		-4.12		0.074	2.829		4	-369.057	746.5	1.26	0.05
47	11.55		-7.709	-8.507	0.069		-4.148	5	-368.027	746.7	1.42	0.046
12	3.324	0.124	-4.117		0.073			4	-369.174	746.8	1.5	0.044
41	4.818				0.066		2.608	3	-370.338	746.9	1.65	0.041
13	6.866			-4.642	0.059			3	-370.349	747	1.67	0.041
16	5.992	0.095	-4.726	-6.107	0.069			5	-368.32	747.3	2.01	0.034
43	7.492		-4.801		0.073		-1.331	4	-369.454	747.3	2.06	0.034
25	1.565				0.064	2.242		3	-370.576	747.4	2.13	0.032
10	3.029	0.090			0.063			3	-370.682	747.6	2.34	0.029
28	-3.897	0.161	-4.504		0.076	3.486		5	-368.506	747.7	2.38	0.028
63	6.119		-7.874	-8.786	0.071	3.047	-3.95	6	-367.486	747.9	2.61	0.025

32	-1.528	0.133	-5.164	-6.391	0.072	3.69		6	-367.559	748	2.75	0.024
45	6.035			-4.25	0.062		2.393	4	-369.911	748.2	2.97	0.021
57	-0.1326				0.068	2.694	2.916	4	-369.934	748.3	3.02	0.021
29	2.582			-4.834	0.060	2.405		4	-370.022	748.5	3.19	0.019
48	8.699	0.098	-7.897	-8.091	0.070		-4.199	6	-367.814	748.5	3.26	0.018
42	1.986	0.098			0.066		2.691	4	-370.128	748.7	3.4	0.017
59	2.437		-4.864		0.075	2.77	-1.067	5	-369.019	748.7	3.41	0.017
44	4.015	0.129	-5.234		0.074		-1.578	5	-369.092	748.8	3.55	0.016
14	4.878	0.066		-4.303	0.059			4	-370.256	748.9	3.66	0.015
26	-2.535	0.116			0.065	2.675		4	-370.293	749	3.73	0.014
64	1.292	0.134	-8.159	-8.264	0.073	3.567	-3.985	7	-367.097	749.4	4.15	0.012
61	0.9205			-4.423	0.064	2.81	2.705	5	-369.468	749.6	4.3	0.011
58	-4.834	0.130			0.069	3.207	3.084	5	-369.574	749.8	4.52	0.01
60	-3.194	0.164	-5.431		0.077	3.425	-1.319	6	-368.448	749.8	4.53	0.01
46	3.723	0.076		-3.847	0.063		2.477	5	-369.788	750.2	4.94	0.008
30	-0.7699	0.092		-4.389	0.061	2.734		5	-369.847	750.3	5.06	0.007
Null model	8.247							1	-374.453	750.9	5.67	0.005
5	9.807			-6.678				2	-373.438	751	5.72	0.005
62	-3.117	0.108		-3.876	0.065	3.222	2.87	6	-369.225	751.4	6.09	0.004
7	11.46		-3.181	-8.212				3	-372.581	751.4	6.14	0.004

3	9.127		-2.16					2	-374.043	752.2	6.93	0.003
33	7.822						1.673	2	-374.252	752.6	7.35	0.002
21	6.483			-6.864		1.893		3	-373.247	752.7	7.47	0.002
17	5.396					1.603		2	-374.318	752.8	7.48	0.002
2	6.103	0.075						2	-374.338	752.8	7.52	0.002
37	9.404			-6.508			1.431	3	-373.288	752.8	7.55	0.002
39	13.96		-5.967	-9.996			-3.714	4	-372.218	752.9	7.58	0.002
23	7.418		-3.389	-8.545		2.365		4	-372.281	753	7.71	0.002
6	8.6	0.041		-6.483				3	-373.404	753.1	7.78	0.002
8	9.786	0.058	-3.254	-7.97				4	-372.513	753.5	8.17	0.002
19	5.849		-2.293			1.874		3	-373.859	754	8.69	0.001
4	6.541	0.093	-2.326					3	-373.868	754	8.71	0.001
35	9.31		-2.395				-0.3404	3	-374.04	754.3	9.05	0.001
49	4.448					1.869	1.867	3	-374.07	754.4	9.12	0.001
34	5.53	0.080					1.733	3	-374.122	754.5	9.22	0.001
53	5.621			-6.691		2.121	1.644	4	-373.051	754.5	9.25	0.001
55	10.02		-6.048	-10.24		2.243	-3.558	5	-371.947	754.5	9.26	0.001
18	2.114	0.094				1.947		3	-374.146	754.5	9.27	0.001
22	4.336	0.060		-6.597		2.103		4	-373.176	754.8	9.5	0.001
38	8.037	0.046		-6.284			1.474	4	-373.246	754.9	9.64	0.001

24	4.458	0.084	-3.523	-8.238	2.676		5	-372.142	754.9	9.65	0.001
40	12.24	0.060	-6.063	-9.758		-3.741	5	-372.144	754.9	9.66	0.001
20	1.804	0.117	-2.533		2.332		4	-373.593	755.6	10.33	0.001
51	5.941		-2.391		1.865	-0.143	4	-373.859	756.1	10.87	0
36	6.775	0.095	-2.68			-0.508	4	-373.86	756.1	10.87	0
50	0.8167	0.102			2.261	1.984	4	-373.866	756.2	10.88	0
56	7.039	0.085	-6.194	-9.934	2.557	-3.573	6	-371.804	756.5	11.24	0
54	3.13	0.068		-6.378	2.371	1.733	5	-372.959	756.6	11.29	0
52	1.977	0.118	-2.743		2.316	-0.3038	5	-373.59	757.8	12.55	0

Table S3: Results of model selection from phylogenetic generalized least squares models relating species functional traits with the occupancy of 99 bird species recorded in 26 gallery forests of an Amazonian savanna landscape, Amapá, Brazil. Models are ranked according to their AICc. Sample-size adjusted AIC (AICc), Akaike differences (Δi) and Akaike weights (wi) are given.

Model	Intercept	Wing morphology	Canopy and Edge	Ground	Habitat specificity	Body mass	Midstory	df	logLik	AIC_c	Δ_i	w_i
13	34.01			-22.75	0.249			3	-480.161	966.6	0	0.106
9	27.84				0.270			2	-481.479	967.1	0.51	0.082
29	13.63			-23.66	0.255	11.44		4	-479.353	967.1	0.56	0.08
47	50.3		-21.35	-35.02	0.264		-21.08	5	-478.532	967.7	1.13	0.06
25	8.652				0.276	10.64		3	-480.797	967.8	1.27	0.056
15	36.3		-5.456	-25	0.260			4	-479.883	968.2	1.62	0.047
63	29.21		-21.99	-36.11	0.273	11.84	-20.31	6	-477.653	968.2	1.64	0.047
31	14.58		-6.745	-26.53	0.269	12.5		5	-478.927	968.5	1.92	0.041
45	35.04			-23.23	0.245		-2.961	4	-480.089	968.6	2.03	0.038
14	29.05	0.166		-21.9	0.251			4	-480.098	968.6	2.05	0.038
10	19.64	0.288			0.271			3	-481.288	968.8	2.25	0.034
30	3.326	0.284		-22.29	0.259	12.45		5	-479.17	969	2.41	0.032
11	28.68		-2.723		0.276			3	-481.408	969.1	2.49	0.03
41	28.38				0.268		-1.784	3	-481.453	969.2	2.58	0.029
26	-5.637	0.405			0.279	12.15		4	-480.426	969.3	2.7	0.027

61	14.68			-23.92	0.253	11.18	-1.721	5	-479.328	969.3	2.73	0.027
27	8.844		-3.726		0.285	11.17		4	-480.664	969.8	3.18	0.022
48	44.07	0.216	-21.76	-34.11	0.267		-21.19	6	-478.423	969.8	3.18	0.022
64	16.67	0.350	-22.73	-34.75	0.278	13.19	-20.4	7	-477.37	970	3.4	0.019
57	8.989				0.275	10.55	-0.5799	4	-480.794	970	3.44	0.019
16	30.41	0.201	-5.762	-24.1	0.263			5	-479.79	970.2	3.65	0.017
32	2.24	0.343	-7.403	-25.16	0.275	13.82		6	-478.663	970.2	3.66	0.017
43	33.61		-9.381		0.281		-9.481	4	-481.097	970.6	4.05	0.014
46	30.35	0.155		-22.42	0.247		-2.79	5	-480.034	970.7	4.14	0.013
12	19.88	0.316	-3.358		0.279			4	-481.182	970.8	4.22	0.013
42	20.24	0.283			0.269		-1.546	4	-481.269	971	4.39	0.012
28	-7.09	0.454	-4.804		0.291	13.02		5	-480.209	971.1	4.49	0.011
62	4.387	0.277		-22.53	0.257	12.23	-1.298	6	-479.157	971.2	4.65	0.01
59	14.07		-9.624		0.289	10.7	-8.459	5	-480.414	971.5	4.9	0.009
58	-5.594	0.405			0.279	12.14	-0.05854	5	-480.426	971.5	4.92	0.009
44	24.32	0.345	-10.54		0.285		-10.14	5	-480.827	972.3	5.72	0.006
60	-2.192	0.476	-11.26		0.296	12.6	-9.188	6	-479.913	972.7	6.16	0.005
5	46.42			-31.34				2	-485.985	976.1	9.52	0.001
21	30.14			-32.25		9.276		3	-485.512	977.3	10.7	0.001
37	48.33			-32.14			-6.758	3	-485.641	977.5	10.96	0

6	44.7	0.059		-31.06			3	-485.978	978.2	11.63	0
7	46.49		-0.1168	-31.4			3	-485.985	978.2	11.65	0
39	59.53		-14.68	-40.73		-19.42	4	-484.974	978.4	11.8	0
Null model	39.1						1	-488.258	978.6	11.98	0
53	33.24			-32.87	8.46	-5.907	4	-485.25	978.9	12.35	0
22	24.84	0.149		-31.59	9.794		4	-485.467	979.4	12.79	0
23	30.4		-0.946	-32.72	9.408		4	-485.504	979.4	12.86	0
38	47.26	0.036		-31.97		-6.725	4	-485.638	979.7	13.13	0
55	44.14		-15	-41.67	8.765	-18.81	5	-484.549	979.7	13.17	0
17	25.03				7.912		2	-487.929	980	13.41	0
33	40.52					-5.562	2	-488.035	980.2	13.62	0
8	44.77	0.060	-0.1919	-31.15			4	-485.978	980.4	13.81	0
3	37.56		3.786				2	-488.132	980.4	13.81	0
2	32.73	0.225					2	-488.156	980.4	13.86	0
40	57.52	0.071	-14.8	-40.45		-19.45	5	-484.963	980.6	14	0
54	28.84	0.122		-32.32	8.903	-5.75	5	-485.221	981.1	14.51	0
24	24.88	0.158	-1.195	-32.15	9.988		5	-485.455	981.6	14.98	0
18	14.19	0.312			9.049		3	-487.739	981.7	15.16	0
49	27.48				7.225	-4.812	3	-487.763	981.8	15.2	0
56	38.48	0.162	-15.28	-41.09	9.36	-18.84	6	-484.496	981.9	15.33	0

19	24.39		3.254	7.527		3	-487.836	981.9	15.35	0
34	34.51	0.211			-5.404	3	-487.945	982.1	15.57	0
35	40.6		-0.1303		-5.672	3	-488.035	982.3	15.75	0
4	32.08	0.199	3.434			3	-488.054	982.4	15.79	0
50	17.12	0.293		8.342	-4.477	4	-487.596	983.6	17.04	0
20	14.52	0.287	2.666	8.644		4	-487.678	983.8	17.21	0
51	27.55		-0.1129	7.225	-4.907	4	-487.763	984	17.38	0
36	34.87	0.215	-0.7727		-6.051	4	-487.943	984.3	17.74	0
52	17.54	0.298	-1.002	8.362	-5.313	5	-487.592	985.8	19.25	0

Table S4: Independent contribution (I) values for landscape and patch-level variables to taxonomic, functional and phylogenetic diversity of bird species recorded in 26 gallery forests of an Amazonian savanna landscape, Amapá, Brazil. Predictor variables in bold indicate that they are significantly related to the response variables (diversity dimension).

Taxonomic Diversity		
	Observed I value	Z score
Average vegetation height	0.72	0.10
Canopy cover	1.17	0.64
Understory foliage density	2.23	1.62
Forest area (%)	1.54	1.09
**Anthropogenic area (%)**	**2.69**	**-2.49**
Savanna area (%)	1.68	1.32
Forest width (m)	0.18	-0.52
Functional Diversity		
Average vegetation height	0.07	-0.66
Canopy cover	0.87	0.35
Understory foliage density	0.64	-0.05
**Forest area (%)**	**2.43**	**2.26**
**Anthropogenic area (%)**	**3.40**	**-2.90**
**Savanna area (%)**	**2.57**	**2.23**
Forest width (m)	0.15	-0.50
Phylogenetic Diversity		
Average vegetation height	0.11	-0.63
Canopy cover	0.48	-0.23
**Understory foliage density**	**2.68**	**-2.60**
**Forest area (%)**	**2.41**	**2.05**
**Anthropogenic area (%)**	**2.65**	**-2.12**
**Savanna area (%)**	**2.28**	**2.15**
Forest width (m)	0.21	-0.49
